# On the matching of top-down knowledge with sensory input in the perception of ambiguous speech

**DOI:** 10.1186/1471-2202-11-67

**Published:** 2010-06-02

**Authors:** C Eulitz, R Hannemann

**Affiliations:** 1 Department of Linguistics, University of Konstanz, Konstanz, Germany

## Abstract

**Background:**

How does the brain repair obliterated speech and cope with acoustically ambivalent situations? A widely discussed possibility is to use top-down information for solving the ambiguity problem. In the case of speech, this may lead to a match of bottom-up sensory input with lexical expectations resulting in resonant states which are reflected in the induced gamma-band activity (GBA).

**Methods:**

In the present EEG study, we compared the subject's pre-attentive GBA responses to obliterated speech segments presented after a series of correct words. The words were a minimal pair in German and differed with respect to the degree of specificity of segmental phonological information.

**Results:**

The induced GBA was larger when the expected lexical information was phonologically fully specified compared to the underspecified condition. Thus, the degree of specificity of phonological information in the mental lexicon correlates with the intensity of the matching process of bottom-up sensory input with lexical information.

**Conclusions:**

These results together with those of a behavioural control experiment support the notion of multi-level mechanisms involved in the repair of deficient speech. The delineated alignment of pre-existing knowledge with sensory input is in accordance with recent ideas about the role of internal forward models in speech perception.

## Background

At the level of speech, most conversations are considerably unclear. How does the brain cope with partly obliterated speech information and how does pre-existing knowledge support these coping-processes? It has been suggested that lexical information can be restored by using top-down lexical knowledge. Here we use the phonemic restoration illusion, where listeners hear spoken words as intact even though parts of them have been replaced by an extraneous sound [1], to study this repair processes in detail.

Given the top-down lexical influences on phonemic processing [2,3] the phonemic restoration illusion can be described as a match of bottom-up sensory input with lexical expectations resulting in resonant neural dynamics [4,5]. Similar, resonant states were first described in studies of feature binding in animals [6]. In humans, such correlates can be measured as an enhancement in the gamma band (GBA) which is discussed among others as a signature of object recognition [7,8] and in relation with several mnemonic processes [9-11]. In language processing, a modulation in GBA was observed for the differentiation between words and pseudowords [12-14] as well as a correlate of merging expected lexical information with degraded speech input [15]. The predominance of the effects over left anterior regions of the brain illustrates the involvement of language competent brain areas.

The present experiment was designed to examine by means of GBA the "filling-in" of phonemic information in the course of phonemic restoration at the pre-attentive level. Whereas the role of top-down repair process for phonemic restoration has been shown in previous ERP experiments for attentive listening to sentences [16,17] a generalization to the preattentive level of processing is still missing. The top-down influence in phonemic restoration at the preattentive level of processing would, however, support the important role of top-down processing for speech perception in general.

We used a roving standard passive oddball paradigm and the point of interest was the detection of a minimal change in the auditory object [18], and the dependency of repair processes of ill-formed speech on the information structure of the preceding auditory object. The preceding object was one of two nouns being a minimal pair in German (*Falte *and *Falke*). The process of merging expected lexical information with the sensory input is expected to result in resonant states [5] which are depicted in the induced GBA. The intensity of repair and thus of the GBA was expected to correlate with the amount of information which has to be aligned.

The assumption about a different amount of information in the lexical representations of the two words used here was based on recent mental lexicon models, which assume abstract and sparse representations of language in the mental lexicon [19,20]. To handle the huge variability of the speech signal during speech perception, these models propose that all nondistinctive and predictable phonological information is not stored in the declarative memory [19,21]. Instead of merely storing all variance in phoneme realizations [22,23], the abstract models assume the underspecification of certain phonemic features in the mental lexicon. Of relevance for the present study was certain place of articulation information which is more sparse for the "t" in *Falte *(underspecified for the [coronal] place of articulation [19,20]) compared to the "k" in *Falke *which is assumed to be phonologically fully specified.

To test whether the specification of phonological details modulate the restoration of phonemes, the induced GBA to the noise-replaced stimuli was investigated. We expected the induced GBA to differ between lexically specified and underspecified information in the precursor. Particularly the induced GBA is expected to be larger in case of a fully specified anticipated phoneme, because more information from the predecessor, has to be aligned and merged with ambiguous auditory input. If this can be found in the present study, the GBA could be interpreted as a correlate of "filling in" the expected and lexically specified information to form a perceivable auditory object out of an ill-formed speech signal.

Alternatively, if the process of phonemic restoration does not differ according to the specification of the phoneme to be restored or if the claim of underspecification in the mental lexicon [19,20] does not hold, no differential modulation of induced GBA should be observable. Moreover, if there is no immediate top-down influence on the phonemic restoration [24,25], no differential modulation of induced GBA should be observable. To substantiate the induced brain activity as a correlate of merging lexical top-down expectancies with obliterated speech input it should be also dissociable from evoked brain activity.

## Methods

### Subjects

Nineteen healthy right-handed monolingual German-speaking volunteers without otolaryngological or neurological diseases participated in this study. Due to bad signal-to-noise ratio three subjects had to be excluded and all further analyses were performed for 16 subjects (eight female; mean age = 23.8 years, standard deviation [SD] = 3.1 years). All participants gave their written consent and received class credits or a small financial bonus. The study was conducted in compliance with the declaration of Helsinki and approved by the ethics committee of the University of Konstanz.

### Stimuli

**Figure 1:**
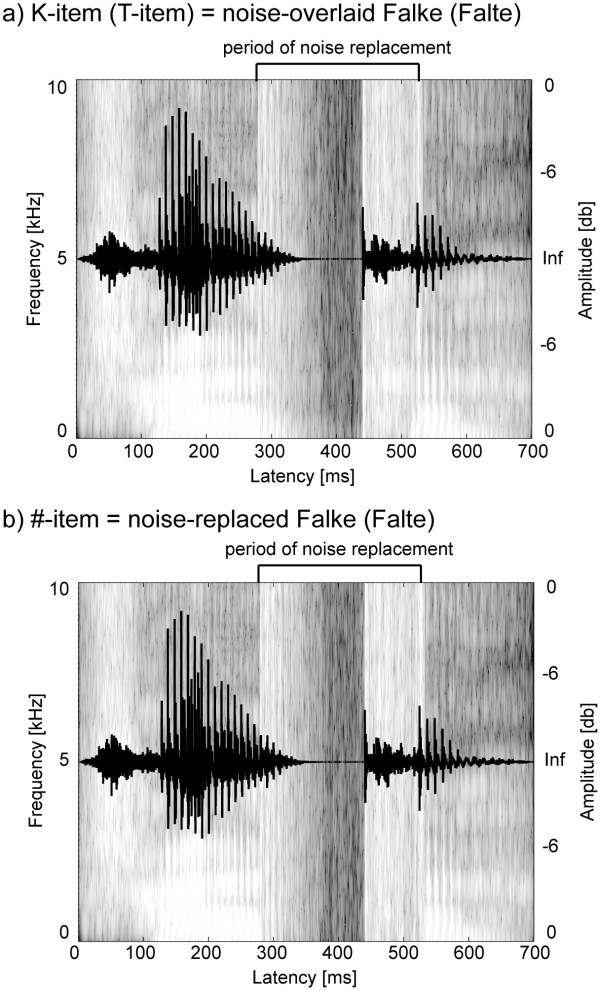
**Stimulus characteristics**. Exemplarily the oscillogram and the spectrogram for a noise-overlaid K-item (a) and a derived noise-replaced #-item (b) are shown.

The experimental stimuli were derived from natural recordings of the minimal pair of the German nouns *Falke *(= hawk) and *Falte *(= fold), which were digitized with 44.1 kHz at 16 bit. For experimental purposes we chose a pair of nouns which were matched for frequency, familiarity and imageability and equalized them further in envelope and second syllable onset using the software package Adobe Audition. Further, we minimized the acoustic difference between both nouns by cross-splicing the first and second syllables with each other resulting in two instances of *Falke *and *Falte*. The latency for cross-splicing was chosen such that no co-articulation of /k/ and /t/ on the phoneme /l/ occurred. This latency was conservatively set at 280 ms post stimulus onset based on results of a separate gating test. (The gating test identified the points of uniqueness for *Falke *at 395 ms and for *Falte *at 370 ms.) To create the noise-replaced items the speech-correlated noise technique [28], which flips the sign of half of the sampling points chosen at random, was used to create a noise from 280 to 520 ms post stimulus onset that maintains the amplitude of the envelope original but has a flattened spectrum. Noise-overlaid items were produced by adding together the critical portions of the replaced and the original versions point for point. Figure 1 illustrates the oscillogram and spectrogram for one exemplar of (a) noise-overlaid and (b) noise-replaced items. This procedure resulted in three stimulus classes for the experiment: each two noise-overlaid Falke (K) and Falte (T) items and four noise-replaced ambiguous (#) items. According to the assumption of a featurally underspecified mental lexicon [19] the critical phoneme in the T-items is underspecified for the [coronal] place of articulation while the featural information for K-items with the [dorsal] place of articulation is fully specified. Finally, all stimuli were normalized for peak amplitude and presented in comfortable loudness (approx. 50 dB SPL) via headphones (Sennheiser PMX 60).

### Acoustic Stimulation

**Figure 2:**
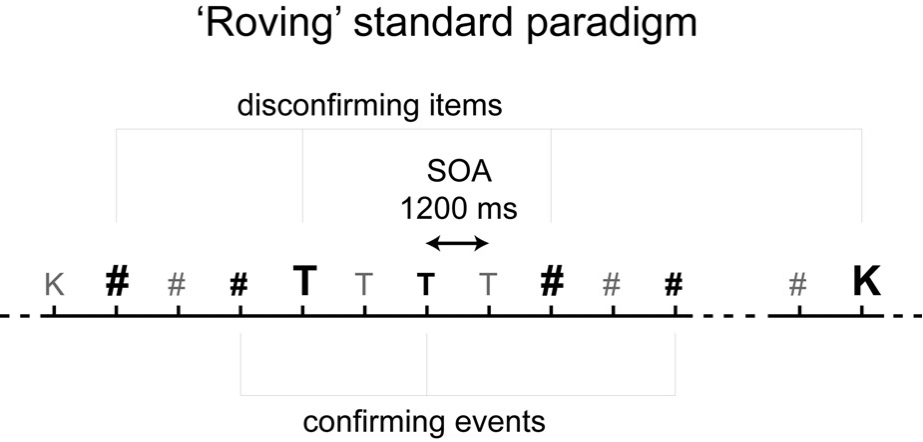
**Illustration of the roving standard stimulation used in this study**. The stimulus sequence consisted of repeatedly changing trains of K, T and #-items (indicated by characters) of a variable number (3 to 8 repetitions) of identical items. Every first item of a train served as disconfirming item (large black) and the respectively third item served as confirming item (small black). The EEG data of disconfirming and confirming items were further analysed. (SOA means stimulus onset asynchrony.)

The present experiment consisted of three experimental blocks of approximately 28 minutes comprising of permanently changing stimulus trains (see Figure 2). One stimulus train consisted of a varying number (from 3 to 8) of K-, T- and #-items. Inside each train of this so called roving standard oddball paradigm [27,28] all items belonged to one stimulus class. Within each train we picked out for further analyses (i) each first item to be the *disconfirming *item with respect to the previous train (corresponds to the deviant in classic oddball paradigms) and (ii) every third item of a train to serve as the so called *confirming *stimulus (corresponds to the standard in classic oddball paradigms). Subsequently, the disconfirming items will be marked as item-type_**1 **_and the confirming items as item-type_**2**_. The number of items per stimulus train as well as the stimulus class of each following train varied randomly through out all three blocks. Overall, this yielded in 12 different conditions (6 disconfirming and 6 confirming) with 120 occurrences each. Moreover, the 6 disconfirming stimulus classes are characterized by having different predecessors (K_1_(t), K_1_(#), T_1_(k), T_1_(#), #_1_(k), #_1_(t)). The corresponding confirming stimulus classes were labeled respectively.

Participants were seated in an electrically shielded and sound attenuated room. During the experiment the subjects were instructed to ignore all stimuli and watched a silent movie. Before the three blocks of passive listening the subjects had to identify the three stimulus classes by pressing a key corresponding to the subjectively heard phoneme at the beginning of the second syllable.

### Data acquisition

**Figure 3:**
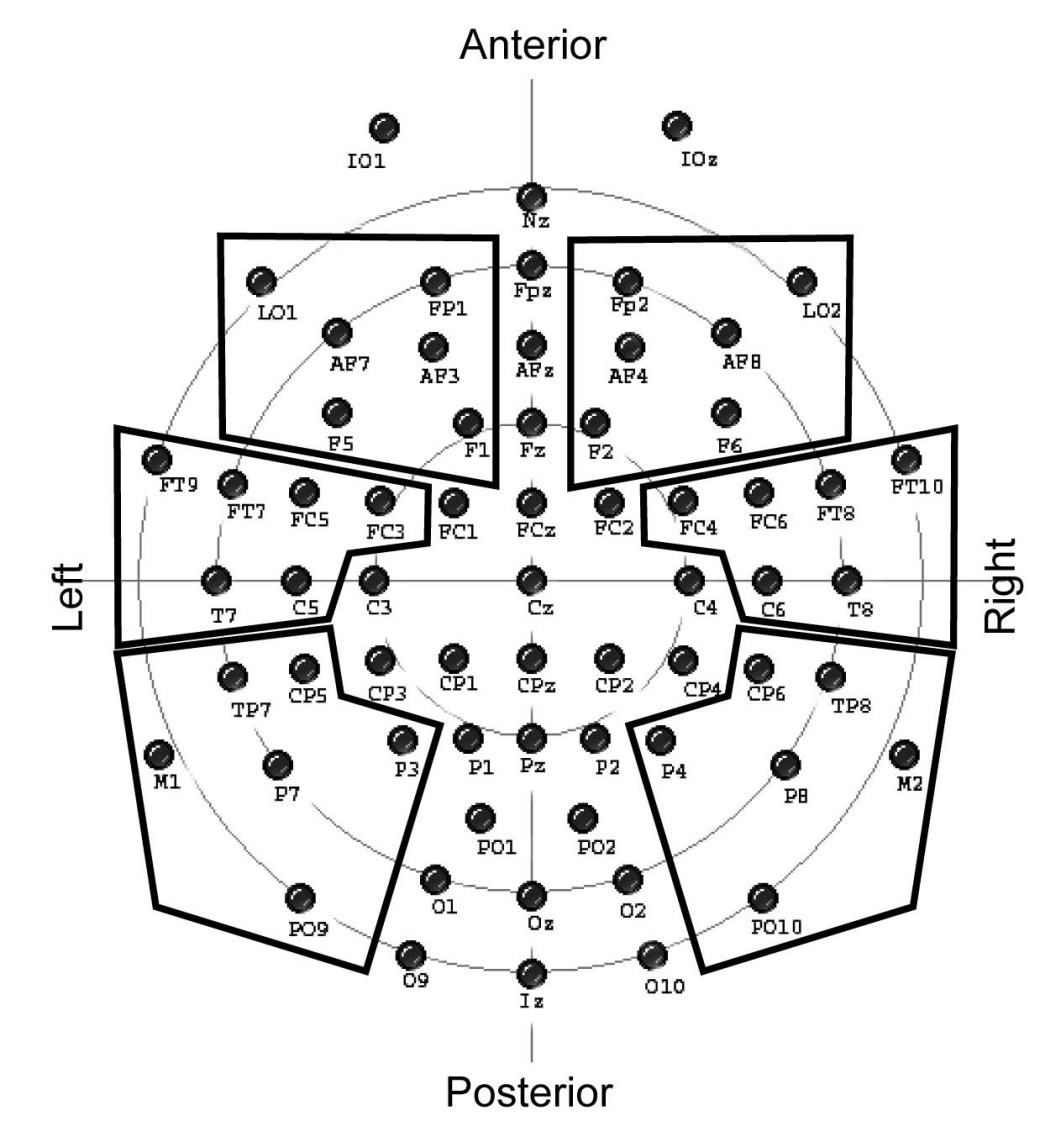
**Electrode montage and groups used for statistical analyses are shown**.

The electroencephalogram (EEG, TMS international, Type Porti S/64) was recorded continuously and digitized with 512 Hz. We used an elastic cap (EASY cap) with 62 scalp electrodes at international 10-10 system locations (average reference) and 2 additional electrodes for controlling eye movements below both eyes (see Figure 3 for a schematic representation for the recording array). The EEG data were band-filtered from 0.1 to 100 Hz. All impedances were kept below 5 kΩ. The continuous EEG was segmented in epochs from 500 ms prior to 1200 ms post stimulus onset. Using the BESA software package, experimental data was corrected for eye artefacts [29] and artefact-flawed epochs were rejected by visual inspection or if epochs exceeded a maximum of 60 μV in amplitude or a gradient of >75 μV.

### Data analysis

To analyze the induced spectral changes in gamma-band activity (GBA; the principle approach was the same as in [15].) in the artefact free epochs from -400 ms to 1000 ms of the disconfirming and confirming items, a wavelet analysis using Morlet wavelets with an m-factor = 7 was performed. By forming a good compromise between frequency and time resolution, this method provides a time-varying magnitude of the signal in each frequency band, leading to time frequency representations of the signal [30]. Then, time by frequency energy is averaged across single trials, allowing one to analyze non-phase-locked frequency components. This method is described in detail elsewhere [31]. In order to achieve a good time and frequency resolution wavelets from 10 to 100 Hz in 2 Hz steps were computed. Next the raw wavelet-data were normalized by computing the relative power change for every time by frequency bin compared to the median of the according baseline which was defined as the latency range from -200 to -100 ms before stimulus onset.

To capture a wide range of cortical sources as well as maintaining a good signal to noise ratio, the mean spectral power of all disconfirming and confirming events was averaged over 6 electrode arrays with 6 electrodes each (Figure 3). Concerning the lack of exact a priori knowledge of latencies and frequencies which might map process of the phonemic restoration in the gamma band, a similar approach as in Hannemann et al. [15] using permutation tests [32] was pursued to compare the differences in spectral power of disconfirming #-items with a K-item as predecessor minus the associated confirming item with the comparable difference having a T-item as predecessor. In the present study these tests were applied to each time-frequency bin from 280 to 1000 ms post stimulus onset for frequencies between 30 and 60 Hz. To make relatively sure that no time-frequency bins passed our criteria by chance, only contiguous bins for at least 30 ms per frequency band which showed a p-value p < 0.01 (uncorrected) were taken into account for further consideration.

Finally a four-way repeated-measures ANOVA Predecessor (K-item vs. T-item) x Expectation (disconfirming vs. confirming) x Hemisphere (left vs. right) x Position (anterior, medial, posterior) was performed on the time-frequency clusters surviving the initial permutation tests to substantiate our findings. For all analyses involving the factor Position, we checked for the violations of the sphericity assumption using Mauchly's criterion, and in case of violations report multivariate testing (using Wilks Lamba) instead. Post-hoc test were only applied to time frequency spots that passed the initial permutation tests. These statistical analyses principally comprised a two way repeated-measures ANOVA Predecessor x Expectation and the belonging t-tests to identify the direction of the predicted modulation in induced GBA.

To dissociate the "filling in" of expected lexical information from a pure phonological conflict depending on the specificity of phoneme representations between the particular predecessor and the pivotal disconfirming noise replaced item, the assessed induced GBA was compared with the mismatch negativity response (MMN) [33,34] which is sensitive to map phonological conflicts in passive oddball paradigms [35,36]. Thus, to ensure that the hypothesized induced GBA is not a mere by-product of a MMN elicited by deviant items (= disconfirming items) interrupting a sequence of repeated standard items, we analyzed the evoked potentials (re-referenced to linked mastoids) with a prestimulus baseline of 100 ms recorded at Fz. Again, we examined the mean amplitude in the latency range identified by the permutations test for the induced GBA using the factorial design as described above.

Further, to differentiate the induced brain activity from evoked brain activity in the gamma band range, we also calculated mean amplitudes of the evoked GBA in the same time by frequency windows as those for the induced GBA and analyzed them using the same factorial design. Finally we also analyzed the induced GBA in higher frequency ranges (76-86 Hz) which are known to reflect electromyographic (EMG) activity for facial and head muscles (in which the peak of the spectral density function of muscular contamination could be expected; [37]) to rule out possible confounds of EMG artefacts [14].

### Behavioural measures

In addition to the EEG study, two behavioural identification experiments were conducted to gain knowledge about the attentive processing of the noise-replaced items. Twelve subjects (seven female; mean age = 24.5 years, standard deviation [SD] = 3.5 years) participated in each of the experiments. They fulfilled the same criteria as the subjects of the EEG study. In the first experiment, which was carried out for exploratory purposes, the subjects had to identify the stimulus-class for all noise-overlaid and noise-replaced items. However, because of the possibility to infer the [coronal] place information from redundant information, larger projection rates in favour of a /t/ percept could be expected for the #-items compared to the /k/ percept, which has a fully specified representation in the mental lexicon instead. Each item was presented six times using the same equipment as for the EEG study. The subjects had to subjectively judge as exact as possible which phoneme has been perceived and respond by pressing the corresponding key on a standard PC keyboard with their right hand.

The experimental design of the second behavioural experiment was made to mimic possible context effects which played a role in the EEG study. Therefore, one experimental trial contained 4 stimuli with the first three items belonging to one item class followed by a fourth item (= target) which could belong to the same or one of the other two item classes. Each of the nine stimulus combinations was presented 24 times which resulted in 216 trials overall. The within trial ISI was 500 ms as in the EEG experiment. After the presentation of the fourth item the subjects had to indicate by button press whether they perceive a K or a T-item at the fourth position as fast and accurately as possible. We hypothesized the reaction times to differ between /k/ and /t/ depending on the specificity of the place of articulation information. Further, the reaction times depicting a successful integration of anticipated and actual sensory input should be longer compared to an unsuccessful unification. To analyze the processing of the noise-replaced items the reaction times (RT) were analyzed by means of a mixed-model ANOVA after cropping the lower and upper 10% percentile.

## Results

### Induced brain responses

**Figure 4:**
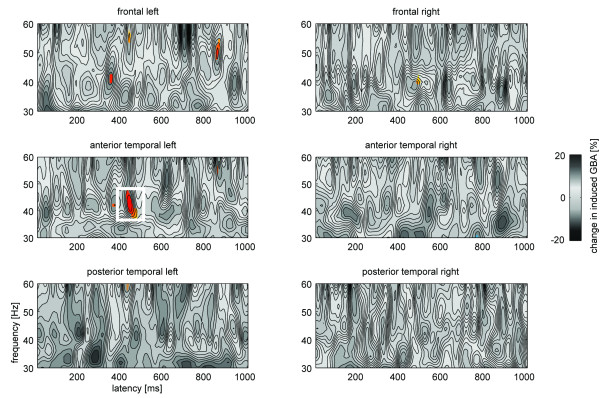
**Grand average time frequency (TF) plots of induced brain activity over six brain regions**. Depicted are the differences of disconfirming #-items over the associated confirming #-items with a precedent K-item compared to those differences with a precedent T-item. Coloured areas reflect TF bins identified as different by a permutation tests with p < 0.01. The framed TF spot further fulfils the continuity criteria and thus constitutes the main finding of this study.

Figure 4 shows the induced brain responses in the gamma band range averaged over the six electrode groups. Depicted are the differences between disconfirming #-items over the respective confirming #-items with a precedent fully specified K-item (= #_1_(k) - #_2_(k)) compared to those differences with a precedent T-item (= #_1_(t) - #_2_(t)) where the critical consonant is underspecified for place of articulation. The time-frequency bins which fulfil the permutation test criteria are shown in colour. As Figure 4 depicts, only one extensive cluster over anterior to medial left hemispheric electrode sites passed the criteria and showed a remarkable difference in the 38 - 44 Hz range from around 430 to 490 ms post stimulus onset. The four way repeated-measures ANOVA in this latency range resulted in a significant Predecessor x Expectation x Hemisphere x Position interaction (F(1,15) = 4.09, p < 0.05). This result confirms the findings of the permutation tests, in that the prominent modulation of the 38 - 44 Hz spectral power is mainly focused on left lateralized anterior to medial electrode sites (see also Figure 4). The changes in induced GBA for the tested time frequency range are summarized in Table 1.

**Table 1:** Mean spectral power for the 430 - 490 ms/38 - 44 Hz range, averaged across six electrode sites and standard error of mean (SEM) in % change for the noise-replaced #-items.

Position	Hemisphere	Expectation created by predecessor	Predecessor K-item		Predecessor T-item	
			
			%GBA change	(± SEM)	%GBA change	(± SEM)
Frontal	Left	Disconfirm	4.29	(2.38)	-3.48	(1.23)
		Confirm	0.29	(1.52)	-2.62	(2.46)
	Right	Disconfirm	2.41	(1.76)	-1.81	(1.70)
		Confirm	-1.81	(1.68)	1.09	(1.66)

Anterior temporal	Left	Disconfirm	**3.69**	**(2.19)**	**-3.59**	**(1.65)**
		Confirm	-3.73	(1.86)	0.26	(1.81)
	Right	Disconfirm	3.77	(3.28)	-1.78	(1.55)
		Confirm	1.82	(1.58)	-2.25	(1.62)

Posterior temporal	Left	Disconfirm	0.51	(1.86)	1.27	(2.00)
		Confirm	1.87	(2.01)	-2.66	(1.93)
	Right	Disconfirm	3.07	(2.36)	-1.49	(1.34)
		Confirm	0.87	(2.52)	-0.64	(1.36)

Figures in bold indicate the main result of the present study.

**Figure 5:**
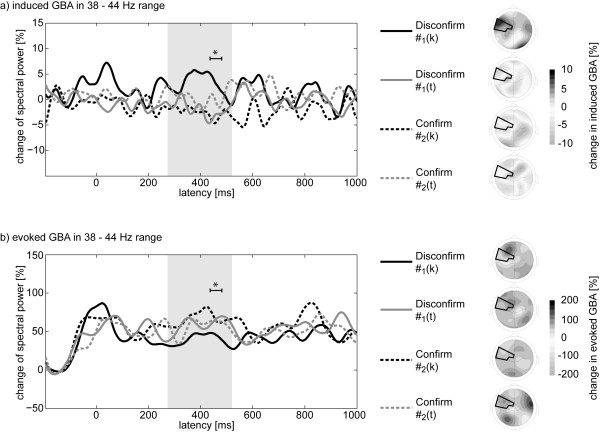
**Time courses of grand average gamma band activity in the 38 - 44 Hz range are shown**. Left: Comparison of the time courses of induced (upper panel) and evoked GBA (lower panel) over left anterior temporal electrode sites. Solid lines represent disconfirming, dashed lines confirming #-items. Black lines picture #-items following K-items and grey lines represent #-items with T-items as predecessor. The underlying grey box pictures the latency range noise-replacement. The starred range always depicts the time course identified by the permutation test. Right: Shown are the topographies of the GBA in the 430 - 490 ms latency range for each #-item respectively. The area of left anterior temporal electrodes sites (cf. Figure 3) is outlined in black. Depicted latency range is the same as indicated by the starred time course on the left (identified by the permutation tests).

Further post-hoc comparisons were performed for the left anterior temporal spot. The time courses and scalp topographies of the induced changes in the 38 - 44 Hz range over this electrode group for the #-items is shown in the upper part of Figure 5. Only the disconfirming #-items following a K-item (#_1_(k), solid black) with a fully specified critical consonant show a substantial increase compared to the other #-items in the latency range of noise replacement (indicated as grey box), especially in the latency range of 430 to 490 ms as identified by the permutation tests. For this time frequency spot, a two way repeated-measures ANOVA revealed a significant Predecessor x Expectation interaction (F(1,15) = 18.39, p < 0.001). Further post-hoc comparisons revealed a significant difference between the disconfirming #-items (t(15) = 2.49, p < 0.05) in favour of a larger value for those #-items which were preceded by a fully specified K-item. No differential modulation was found for the confirming #-items (t(15) = 1.61, p > 0.1). Additionally post-hoc tests showed significant differences between the disconfirming and the confirming #-items following K-items (t(15) = 2.64, p < 0.05) as well as T-items (t(15) = 2.43, p < 0.05). The mean values indicated a positive difference in spectral power if the K-items were in predecessor position and a negative difference if the T-items were in predecessor position. Despite the opposing directionality in the evolution of the spectral power for the #-items following K and T-items it is important to note, that the main modulation in induced GBA was observed for the disconfirming #-items whereas the confirming #-items did not differ.

As Figure 5a indicates, the modulation in 38 - 44 Hz spectral power might last longer than the initial permutation test suggested. For the latency range from 350 to 490 ms the Predecessor x Expectation interaction was also significant (F(1,15) = 12.25, p < 0.001) with post-hoc t-tests showing significant larger values in spectral power for #_1_(k) compared to #_1_(t) items (t(15) = 2.96, p < 0.01) and significant differences between #_1_(k) and #_2_(k) items (t(15) = 2.86, p < 0.05). All other post-hoc analyses revealed no significant differences (p > 0.2) for this latency range.

Although our predictions concerning the induced spectral changes were only specific to the "filling in" of expected lexical information as processing step to build up a percept of a phoneme (which are expected to appear first after onset of the noise replacement begins) the time course of the induced 38 - 44 Hz changes in Figure 5a points to another modulation around the onset of the #-items (-50 - 100 ms). However, the corresponding ANOVA showed neither a significant Predecessor x Position interaction (F(1,15) = 1.87, p > 0.1) nor any main effect (all F < 1.5, p > 0.2) for the 38 - 44 Hz range and reinforces therefore the non-result of the permutation tests for this latency range.

### Analyses of evoked gamma band responses and control for possible EMG confounds

To ascertain that our results indeed reflect modulations of induced brain activity, we post-hoc analyzed the evoked brain activity for the same time and frequency range. Figure 5 contrasts the time course of the induced and evoked spectral changes in the 38 - 44 Hz range for left anterior medial temporal electrode sites. As exemplified only the induced spectral changes showed a modulation on the disconfirming #-items with larger values for the #_1_(k) items compared to #_1_(t) items whereas the evoked spectral changes exposed no comparable modulation pattern. Statistical analyses analogous to the analyses for the induced brain activity revealed neither a four-way interaction (F(1,15) < 1, p > 0.4) nor any main effect or interaction for the left anterior temporal electrode sites (all F < 1).

Finally, to test for possible EMG artefacts which might be correlated with the induced result in the 38 - 44 Hz range, a four-way ANOVA testing the 76 - 86 Hz range [37] yielded no comparable results for the latency range identified by the permutation tests, especially no Predecessor x Expectation x Hemisphere x Position interaction (F(1,15) = 1.84, p > 0.1).

### Differentiation of induced brain responses from ERP results at Fz

**Figure 6:**
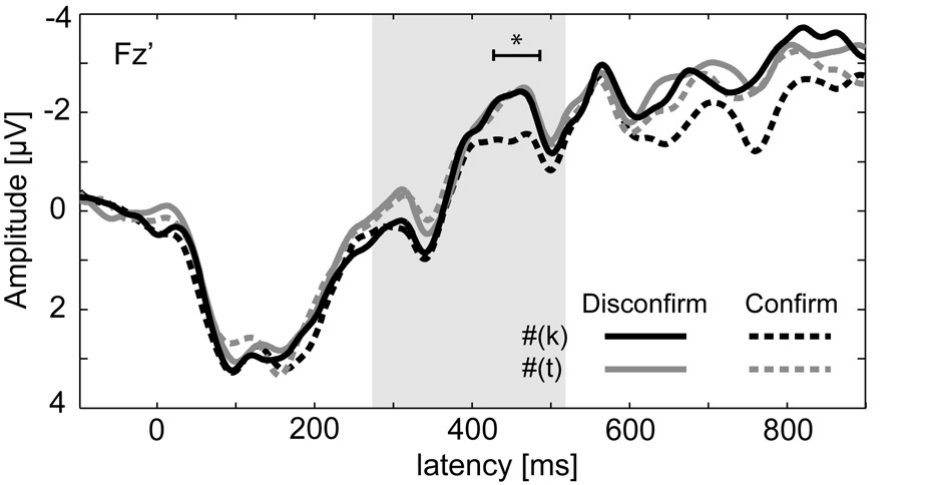
**Evoked potentials at Fz-electrode position is shown for #-items**. Solid lines represent disconfirming and dashed lines confirming #-items. Those #-items following K-items are shown in black and #-items with T-item as predecessor are depicted in grey. The underlying grey box shows the latency range of noise-replacement. The starred range depicts the analyzed time course as identified by the permutation tests for the induced GBA.

Figure 6 depicts the re-referenced (Fz) evoked potentials (ERP) for the disconfirming and confirming #-items following either stimulus trains of fully specified K or underspecified T-items recorded at Fz. In the latency range identified with the permutation tests for the induced brain activity (430 - 490 ms) all #-items except the #_2_(k)-items show the same activity pattern. An ANOVA analogous to the induced brain responses showed only a barely significant main effect of Expectation (F(1,15) = 4.64, p < 0.048). The mean values of amplitude point to a stronger negativity for the disconfirming #-items (mean = -2.23 μV) compared to the confirming #-items (mean = -1.79 μV). Notably, statistical analyses revealed neither a Predecessor x Expectation interaction (F(1,15) = 2.44, p > 0.1) nor a main effect of Predecessor (F(1,15) = 1.91, p > 0.1) in this latency range, although Figure 6 might suggest the opposite.

### Behavioural measures

As shown in Table 2, the results of the first identification experiment indicate that despite some miscomprehensions the noise-overlaid segments in the K-items and T-items were perceived significantly above chance level as /k/ (72.4%) or /t/ (79.2%) respectively. For the #-items the data revealed a tendency to perceive the noise-replaced segment as /t/ (34.8%) or /k/ (27.5%) rather than anything else. These results are very similar compared to the results from the short initial exploration at the beginning of the passive listening in the roving oddball task. Here the subjects reported perceiving the noise-replaced part as /t/ (47.3%), /k/ (17.8%) rather than any response else.

**Table 2:** Mean identification rates for the K, T and #-items across 12 subjects of the first behavioural experiment.

		Rate of Keystroke [%]		
		K - key	T - key	Remaining keys
Item-class	K-Items	72.44	23.08	4.48
	T-items	18.18	79.22	2.60
	#-items	27.48	34.82	37.70

The results of the second behavioural experiment are summed up in Table 3. With respect to the influence of the predecessor context on the perception of the #-items, there is a clear preference to interpret the #-items as a /t/ percept independently of the context. This is reflected in a significant main effect of Percept (F(1,11) = 17.24, p < 0.01) for the projection rate. However, the reaction time data indicate an influence of the predecessor. Here, subjects go faster for the opposite percept relative to the predecessor. This pattern results in a significant Predecessor x Percept interaction (F(1,457) = 7.99, p < 0.01). However, the post-hoc test for the #-items which were interpreted like the predecessor revealed no difference in reaction time between the /t/ and /k/ percepts (F(1,11) < 1, p > 0.1)

**Table 3:** Reaction times (RT) and the projection rate (PR) of #-items onto /k/ and /t/ percepts in different contexts are summarized for the second behavioural experiment.

			K - Percept		T - Percept	
	pre-decessor	disconf. item	RT in [ms]	PR in [%]	RT in [ms]	PR in [%]
Item Sequence	K-Items	#-Item	1056.7	28.5	987.4	71.5
	T-Items	#-Item	991.3	26.9	1037.9	73.1
	#-Items	#-Item	1014.4	33.3	1031.7	66.7

## Discussion

To gain a better understanding of how the brain copes with acoustically ambivalent situations the present study was set out to shed light on the brain mechanisms underlying the repair of fragmentary speech information. Particularly the study investigated the role of lexical specification of phonological details in the mental lexicon and its impact on the phonemic restoration illusion. In order to prevent influences of attention or decision making processes on the phonemic restoration the illusion was investigated by means of a passive oddball paradigm in which the subjects were instructed to ignore the auditory stimuli. In doing so, the study goes beyond previous EEG studies with active tasks [16,17]. To monitor the processing of ambivalent sensory input under the influence of differential top-down mediated expectations of phonemic features we examined the induced GBA. If the fine structure of phonological information in the mental lexicon may play a significant role in the phonemic restoration, we hypothesized a differential modulation in the induced GBA depending on the specificity of the place of articulation of the phoneme to be restored. Our results for the left anterior electrode sites clearly support this assumption. In the latency range of the to-be-expected phoneme for the disconfirming #-items we observed larger values of induced GBA if the expected phoneme was specified for the feature place of articulation (K-item) compared to the underspecified expectation (T-item). These larger values of induced GBA were most pronounced between 430 and 490 ms in the 38 - 44 Hz range. Importantly, there was no differential modulation in induced GBA for the confirming #-items. As Figure 5 illustrates the evoked GBA showed no comparable effects, neither for the disconfirming nor the confirming #-items.

The topography of the effect is similar as in Hannemann et al. [15]. As there, the modulation of induced GBA over left anterior temporal electrode sites can be interpreted as a correlate for a match of bottom-up sensory input with lexical expectations resulting in resonant neural dynamics [4,5].

The present results showing the differential modulation of induced GBA in the restoration illusion is also interesting from another point of view. The difference was predicted based on a speech perception model which assumes underspecified mental representations of certain features of the sound structure. According to the featurally underspecified lexicon theory [19,20] the critical phoneme in the K-items possesses a full featural specification for the [dorsal] place of articulation while the [coronal] place of articulation for T-items is underspecified. Thus, while the repeatedly presented K-items establish an expectation of a specified place of articulation in the critical phoneme, the T-item cannot build up such specific expectations based on specified featural information in the mental lexicon. This difference was reflected in our GBA results and would not have been predicted by other speech perception models [22,23]. Moreover, the pre-attentive modulation of induced GBA is further evidence for an immediate lexical top-down support in the phonemic restoration [2] and generally in the perception of speech in difficult auditory environments. Thus, the present results suggest a more extensive and immediate top-down influence on repair processes in speech perception as claimed by more autonomous views on speech perception [24,25].

It is well established that signatures in GBA can differentiate between words and pseudowords [12,13]. As all #-items were acoustically identical (and in a strict sense all pseudowords) this known difference should maximally lead to a main effect of Expectation and does therefore not explain the present results. Thus models favoring strictly bottom-up processes in speech perception [38] cannot account for the observed differential modulation in induced GBA, especially because all #-items were physically equal and there is no post-perceptual decision making process which might have influenced the GBA.

According to Pulvermuller et al. [39], activity in higher frequency bands contains information about semantic features of words, i.e. it shows differential topographies between verbs and nouns in a lexical decision task. Recently an intracerebral EEG study observed modulations in evoked GBA in a visual semantic decision task [40]. Following this argumentation it might be possible, that the observed modulation in induced GBA in the present study is caused by different semantic instead of phonological expectations. As both words which create the expectation for the disconfirming #-items are nouns, were matched for frequency and the observed effect cover different frequency bands this interpretation seems rather unlikely. Nevertheless it can't be absolutely ruled out that the larger value of induced GBA for #_1_(k)-items compared to #_1_(t)-items is at least partly due to a differential semantic expectation.

With respect to the findings of Eulitz et al. [36] the suspending of repeated presentation of fully specified with underspecified items lead to larger phonological conflicts mapped in differential MMNs than vice versa. Thus, if the present modulation in induced GBA in favor to the #_1_(k)-items is due to that kind of phonological conflict the MMN should also show a differentiating pattern between the disconfirming expectations of specified and underspecified items. As we found only a general difference between disconfirming and confirming #-items which was independent from the predecessor context the present results cannot be explained by variable strength of phonological conflicts, at least in the present latency range of 430 - 490 ms.

The results of the behavioural experiments support and extend our interpretation of the observed gamma-band modulation during the processing of the #-items. When attending the stimuli, the pattern of results is different compared to the pre-attentive processing of #-items. Without context, as in the first behavioural experiment, the subjects showed a preference towards perceiving a /t/ over a /k/ and all other possible phonemes. The same pattern of results was obtained in the pre-experimental exploration. This identification bias toward /t/ was replicated for the projection rates in the second behavioural experiment. Due to the lack of alternatives in this choice task, this bias was even more pronounced. This bias can be interpreted in two ways: (i) The [coronal] place of articulation is regarded as the default place of articulation by phonologists [41]. In absence of any information indicating a specification of the place of articulation in the mental lexicon, the subjects therefore showed a preference towards perceiving a /t/ for the ambiguous acoustics in the #-items. (ii) It might be also due to the spectral characteristics of the noise replacing the critical consonant, which is spectrally slightly more similar to a /t/ compared to a /k/ [42]. Interestingly, reaction time data of the second behavioural experiment indicated context effects. When subjects decided that the actual #-item was the same as the predecessors, the reaction times to these #-items was significantly longer compared to the inexpedient response. The longer RT seems to indicate a more complex decision and evaluation process, which is required to align the anticipated phonemic information and the sensory input. Under attentive processing conditions, this RT effect is independent of the specification of featural information in the mental lexicon.

According to that, the modulation in induced GBA in favour of the #_1_(k)-items and the prolonged RT enlighten differential aspects of the phonemic restoration illusion. Both describe the matching processes of deficient sensory input and anticipated phonemic information. But, as the behavioural data is generally influenced by external factors, i.e. task formulations and attention etc., the pre-attentive EEG data is free of such influences and thus yield additional insights on the influence of the fine structure of the mental lexicon on this matching process. However, the present results are only a first step towards a comprehensive understanding of the influence of the specificity of phonemes in the mental lexicon on repair processes in speech perception. Further studies investigating other features in German and other languages are crucial to allow for a general comprehension of speech perception under natural and noisy conditions.

## Conclusion

In sum, the current study evinces for the first time a direct correlate for a top-down modulated "filling in" in the phonemic restoration illusion without relying on redundant sentential information. The present induced brain responses again reveal clear evidence for a left lateralized functional network in matching expected lexical information with sketchy sensory input [15] to form a coherent auditory object [18]. Further, they demonstrate the influence of the fine structure in the mental lexicon on top-down modulated speech perception processes and are in line with current cortical models of auditory word recognition [43]. Moreover, the delineated alignment of lexical expectancies with sensory input is in accordance with recent ideas that speech perception is facilitated by internal forward models [44]. Thus it serves as prerequisite for speech and more generally for conscious object perception [7]. Finally the current results experimentally show that the human ability to comprehend speech even pre-attentively and under much compromised conditions (i.e. restoring missing phonemes) relies on the immediate interaction of lexical expectancies (i.e. top-down) and the acoustical input. These interactions can be examined by means of induced GBA.

## Competing interests

The authors R. Hannemann and C. Eulitz declare that they have no competing interests.

## Authors' contributions

RH prepared the stimuli, designed and implemented the current study, was responsible for collecting, analyzing and interpreting the data and drafted the manuscript. CE made substantial contributions to conception and the experimental design, and supervised the data analyses. CE was intensely involved in interpreting the data and drafting the manuscript. Final revisions were made by CE. All authors read and approved the final manuscript.
